# Rural–urban variation in insecticide-treated net utilization among pregnant women: evidence from 2018 Nigeria Demographic and Health Survey

**DOI:** 10.1186/s12936-020-03481-5

**Published:** 2020-11-11

**Authors:** Edward Kwabena Ameyaw, Kenneth Setorwu Adde, Shadrach Dare, Sanni Yaya

**Affiliations:** 1 School of Public Health, Faculty of Health, University of Technology Sydney, Sydney, NSW Australia; 2 Department of Population and Health, College of Humanities and Legal Studies, University of Cape Coast, Cape Coast, Ghana; 3 Mother and Infant Research Unit, University of Dundee, Dundee, UK; 4 School of International Development and Global Studies, University of Ottawa, Ottawa, Canada; 5 The George Institute for Global Health, The Imperial College London, London, United Kingdom

**Keywords:** Pregnant women, Insecticide treated net, ITN, Maternal health, Malaria, Nigeria

## Abstract

**Background:**

In 2018, Nigeria accounted for the highest prevalence of malaria worldwide. Pregnant women and children under five years bear the highest risk of malaria. Geographical factors affect utilization of insecticide-treated nets (ITN), yet existing literature have paid little attention to the rural–urban dimension of ITN utilization in Nigeria. This study aimed at investigating the rural–urban variation in ITN utilization among pregnant women in Nigeria using data from the 2018 Demographic and Health Survey.

**Methods:**

A total of 2909 pregnant women were included in the study. The prevalence of ITN utilization for rural and urban pregnant women of Nigeria were presented with descriptive statistics. Chi-square test was employed to assess the association between residence, socio-demographic characteristics and ITN utilization at 95% level of significance. Subsequently, binary logistic regression was used to assess the influence of residence on ITN utilization.

**Results:**

Eight out of ten of the rural residents utilized ITN (86.1%) compared with 74.1% among urban residents. Relative to urban pregnant women, those in rural Nigeria had higher odds of utilizing ITNs both in the crude [cOR = 2.17, CI = 1.66–2.84] and adjusted models [aOR = 1.18, CI = 1.05–1.24]. Pregnant women aged 40–44 had lower odds of ITN utilization compared to those aged 15–19 [aOR = 0.63, CI = 0.44–0.92]. Poorer pregnant women had higher odds of ITN utilization compared with poorest pregnant women [aOR = 1.09, CI = 1.04–1.32]. Across regions, those in the south [aOR = 0.26, CI = 0.14–0.49] and south-west [aOR = 0.29, CI = 0.16–0.54] had lower odds of ITN use compared to their counterparts in the north-west region.

**Conclusion:**

The high use of ITNs among pregnant women in Nigeria may be due to the prioritization of rural communities by previous interventions. This is a dimension worth considering to enhance the attainment of the national anti-malarial initiatives. Since possession of ITN is not a guarantee for utilization, women in urban locations need constant reminder of ITN use through messages delivered at ANC and radio advertisements. Moreover, subsequent mass ITN campaigns ought to take cognizance of variations ITN use across regions and pragmatic steps be taken to increase the availability of ITN in households since there is a moderately high use in households with at least one ITN in Nigeria.

## Background

Malaria continues to pose a major threat to public health worldwide as 91 countries reported a total of 228 million cases in 2018 with 405,000 deaths [1]. The latest World Malaria Report reported that sub-Saharan Africa (SSA) accounts for 93% of all malaria cases and 94% malaria deaths [1]. Although malaria affects persons of all ages and gender, it is phenomenal among pregnant women and children under 5 years [2]. Pregnant women are prone to malaria largely because of declined immunity and conducive milieus for malaria parasites created by the placenta [3]. Children under the age of five years are also prone to severe forms of malaria as they have lost maternal immunity and yet to develop specific immunity towards infections [4].

Malaria during pregnancy constitutes a substantial risk for the mother, her fetus and newborn [5]. Compared to non-pregnant women, pregnant women have a three-fold increased risk of malaria opportunistic diseases [6]. It is also estimated that the malaria related mortality rate among pregnant women is about 50% higher [6]. Malaria increases risks of intra-uterine growth retardation, low birth weight and neonatal deaths [7, 8].

In 2018, Nigeria accounted for the highest prevalence of malaria worldwide by recording 25% of all malaria cases, and 24% of all malaria deaths worldwide [1]. This represents a 6% (3.2 million) increase in the absolute number of cases from 2017. Malaria remains endemic in Nigeria with pregnant women and children under five years of age bearing the highest risk [9]. Pregnant women and mothers of children under five years sometimes lack comprehensive knowledge about malaria [10] but may be aware of the malaria disease [11].

Given the menace of malaria, the Federal Government of Nigeria has since 2001 implemented four National Strategic Malaria Strategic Plans (NSMSP) (2001–2005; 2006–2010; 2009–2013 and 2014–2020). In the year 2000, the Roll Back Malaria (RBM) Partnership was initiated in Abuja, Nigeria where it was recommended that pregnant women sleep under insecticide-treated nets (ITN) [12]. The aim of the current NSMSP, under the National Malaria Elimination Programme (NMEP), is to reduce the incidence rate of malaria to less than 5000 per 100,000 persons and reduce deaths attributable to malaria to zero by 2020. The first of seven objectives of this goal is to provide at least 80% of the targeted population with appropriate preventive measures by 2020, including a universal access to ITNs [13]. ITN ownership in Nigeria has since increased from 8% in 2008 to 61% in 2018 [14, 15]. There are two points to note here; firstly, ITN ownership in Nigeria is likely to vary by region, place of residence or other maternal demographic characteristics [16, 17]. Secondly, ITN ownership does not necessarily imply utilization, which is more related to malaria prevention. In a peri-urban city in Nigeria, Tobin-West and Kanu [18] observed that while 49% of surveyed women owned ITN, only 18% used it consistently.

Moreover, a number of ITN campaigns have been initiated in the country. These initiatives have targeted rural and urban locations and across the various states. For instance, the NMEP adopted a mixed-model approach for ITN distribution and this included free mass distribution campaigns and continuous distribution of ITNs [19]. The dominant channels for distribution included community-based distribution and integrated maternal, newborn and child health weeks as well as antenatal clinics. Through this, 77% households within the catchment locations received ITNs [19]. Community-based distribution was piloted around 2013 but was not scaled up. State specific mass campaigns include the mass ITN replacement campaign in Zamfara State, northwest Nigeria [20], central Nigeria based integrated insecticide-treated bed net distribution programme [21] and the universal insecticide-treated net campaigns in northern Nigeria [22]. In the case of Zamfara State, mass ITN replacement exercise took place and over two million nets were distributed in all. The National Malaria Control Programme (NMCP) Strategic Plan 2009–2013 targeted children and aimed at ensuring that at least 80% of children under five and pregnant women consistently use ITN. Other similar interventions focused on both rural and urban locations in twenty-one states using mass media campaigns through national radio and television stations [23].

Few studies have focused on the utilization of ITN among pregnant women although they constitute one of the high-risk groups, and the findings have been inconsistent [24–26]. Studies in Nigeria have only considered rural–urban variation in ITN utilization as a covariate and have reported contradictory findings. For instance, whilst insignificant association has been found with higher odds for rural women [27], insignificant lower odds of among rural women has also been reported [22]. Meanwhile Ankomah et al. [28] noted a significant association with higher odds of ITN use among urban residents in Nigeria [28]. Rural–urban variation in ITN use in Nigeria has not been granted the requisite audience in the literature and given that geographical and seasonal variations strongly influence malaria and ITN use [29], investigating rural–urban dimension of ITN use is fundamental in guiding policies and targeted actions on malaria programme interventions. This study, therefore, examined the urban–rural variation in the utilization of ITN among pregnant women using data from the 2018 Nigeria Demographic and Health Survey (NDHS). This is important for tracking and understanding the progress of the Roll Back Malaria (RBM) programme as well as other malaria control programmes. The findings would help improve existing malaria control strategies as well. An understanding of the distribution or variation of ITN utilization is essential to planning targeted public health interventions and understanding progress towards national goals. The findings would also help improve existing malaria control strategies.

## Methods

### Data source

Data from the women’s recode file of the 2018 NDHS was used [14]. The survey targeted women aged 15–49 years. The 2018 NDHS aimed at providing current estimates of basic demographic and health indicators. It captured information on ITN ownership and utilization, fertility, awareness and utilization of family planning methods and other essential maternal and child health indicators. In this study, data on a sub-sample of women who reported that they were pregnant during the survey was analysed.

Information gathered through the survey are intended to help programme managers and policy makers in assessing and developing programmes and interventions for enhancing the health status of Nigerian people. The National Population Commission (NPC) implemented the survey whilst ICF offered technical assistance through the Demographic and Health Survey (DHS) Programme. The data collection occurred between August 14 and December 29, 2018. The sampling frame for the study was developed from the 2006 Population and Housing Census of Nigeria. The 2018 NDHS was executed through a two-stage stratified sampling approach. The survey achieved 99% response rate. Full description of the sampling approach and other methodological issues are elaborated in the report [14].

### Study variables

#### Outcome variable

The principal outcome variable for this study was ITN utilization. ITNs were listed in the household of all women who participated in the survey and they responded to the question on whether the ITN has been used and by whom. The response of women who slept under ITN the night before were coded yes as ‘1′ or no ‘0.’ Following the same coding, our outcome variable was sleeping under ITN (maintained as 1), whilst not sleeping under ITN was captioned as otherwise ‘0′. Only responses from women who indicated that they were pregnant at the time of the survey and had at least one ITN in their households were included.

#### Independent variable

Residence (urban or rural) was the main independent variable for the study. The DHS conceptualizes urban areas to comprise large cities (e.g. capital cities and cities having more than 1 million population), relatively small cities with over 50,000 population; All rural settings are considered as countryside residences [30]. This independent variable was not considered per chance, instead, the selection was informed by debate on how location or environmental context informs ITN utilization [31–33]. Similarly, some covariates were included based on theoretical relevance and conclusions drawn about their association with ITN utilization [34–36]. These variables are age, education, wealth quintile, religion, marital status, health decision-making, pregnancy intendness or otherwise, health insurance and frequency of engagement with mass media (radio, television, newspaper) and region.

#### Analytical technique

The analysis was done using STATA version 13. It begun with descriptive computation of ITN utilization with respect to residence and the covariates. These were presented as frequencies and percentages. Chi-square tests were conducted to explore the level of significance between residence, covariates and ITN utilization at 5% margin of error (Table 1). In the next step, binary logistic regression analysis was carried out to determine the influence of residence on ITN utilization among the pregnant women as shown in the first model (Model I in Table 2). The results of this model was presented as crude odds ratios (cOR) with their corresponding 95% confidence intervals. The effect of covariates was explored to ascertain the net effect of residence on ITN utilization in the second model (Model II in Table 2) where adjusted odds ratios (aOR) were reported. Normative categories were chosen as reference groups for the independent variables [37]. Sample weight was applied whilst computing the frequencies and percentages so that we could obtain results that are representative at the national and domain levels [14]. STATA’s survey command (SVY) was used in the regression models to cater for the complex sampling procedure of the survey. Multicollinearity was assessed among the co-variates with Variance Inflation Factor (VIF) and it was evident that no multicollinearity existed (maximum VIF = 2.33; minimum VIF = 1.02, mean VIF = 1.49).

**Table 1:** Residential status, socio-demographic characteristics and ITN utilization (N = 2909)

Variable	Weighted N	Percentage (%)	Slept under ITN	
			Yes (%)	No (%)
Residence				
Urban	898	30.9	74.1	25.9
Rural	2011	69.1	86.1	13.9
Age, years				
15–19	387	13.3	87.2	12.8
20–24	667	22.9	84.2	15.8
25–29	784	27.0	80.9	19.1
30–34	560	19.3	83.8	16.2
35–39	343	11.8	76.4	23.6
40–44	123	4.2	77.2	22.8
45–49	45	1.6	83.0	17.0
Education				
No education	1600	55.0	88.1	11.9
Primary	371	12.8	80.1	19.9
Secondary	746	25.7	73.6	26.4
Higher	191	6.6	73.3	26.7
Wealth Quintile				
Poorest	747	25.7	88.5	11.5
Poorer	791	27.1	88.9	11.1
Middle	600	20.6	83.3	16.7
Richer	426	14.6	68.5	31.5
Richest	345	11.8	69.9	30.1
Religion				
Christianity	754	25.9	74.3	25.7
Islam	2138	73.5	85.2	14.8
Traditionalist/Other	17	0.6	91.5	8.5
Marital status				
Married	2836	97.5	82.6	17.4
Cohabiting	73	2.5	74.9	25.1
Health decision making				
Alone	214	7.3	78.5	21.5
With partner	683	23.5	75.2	24.8
Husband/partner alone	1997	68.7	85.3	14.7
Other person	14	0.5	80.0	20.0
Wanted current pregnancy				
Not at all	65	2.2	70.3	29.7
Then	2588	89.0	83.8	16.2
Later	256	8.8	71.7	28.3
Health insurance				
No	2827	97.2	82.5	17.5
Yes	81	2.8	79.2	20.8
Frequency of listening to radio				
Not at all	1562	53.7	85.2	14.8
Less than once a week	587	20.2	80.7	19.3
At least once a week	760	26.1	77.9	22.1
Frequency of watching T.V				
Not at all	1915	65.8	87.4	12.6
Less than once a week	369	12.7	77.1	22.9
At least once a week	624	21.5	70.2	29.8
Frequency of reading Newspaper				
Not at all	2664	91.6	82.9	17.1
Less than once a week	175	6.0	74.0	26.0
At least once a week	70	2.4	84.8	15.2
Region				
North central	373	12.8	79.1	20.9
North east	556	19.1	82.0	18.0
North west	1440	49.5	89.4	10.6
South east	183	6.3	74.6	25.4
South south	135	4.6	63.3	36.7
South west	220	7.6	61.3	38.7

*χ*^*2*^ Chi-square, *df* degree of freedom

**Table 2:** Binary Logistic regression on residential status, socio-demographic characteristics and ITN utilization

Variable	Model I		Model II	
	cOR 95% CI		aOR 95% CI	
Residence				
Urban	1	[1]	1	[1]
Rural	2.17***	[1.66–2.84]	1.18**	[1.05–1.24]
Age				
15–19			1	[1]
20–24			1.10	[0.81–1.52]
25–29			0.98	[0.72–1.30]
30–34			1.26	[0.87–1.43]
35–39			0.72	[0.58–1.28]
40–44			0.63**	[0.44–0.92]
45–49			0.70	[0.51–2.08]
Education				
No education			1	[1]
Primary			0.80	[0.70–1.19]
Secondary			0.76	[0.64–1.20]
Higher			0.86	[0.72–1.43]
Wealth Quintile				
Poorest			1	[1]
Poorer			1.09**	[1.04–1.32]
Middle			0.90	[0.60–1.36]
Richer			0.53	[0.43–1.86]
Richest			0.63	[0.36–1.10]
Religion				
Christianity			1	[1]
Islam			0.71	[0.47–1.07]
Traditionalist/Other			1.21	[0.27–5.45]
Marital Status				
Married			1	[1]
Cohabiting			1.45	[0.74–2.83]
Health Decision Making				
Alone			0.84	[0.55–1.28]
With Partner			0.92	[0.68–1.22]
Husband/Partner Alone			1	[1]
Other person			0.64	[0.18–2.26]
Wanted current pregnancy				
Not at all			0.87	[0.47–1.60]
Then			1	[1]
Later			0.66*	[0.46–0.97]
Frequency of listening to radio				
Not at all			1	[1]
Less than once a week			1.14	[0.80–1.62]
At least once a week			1.32**	[1.07–1.74]
Frequency of watching T.V				
Not at all			1	[1]
Less than once a week			0.83	[0.56–1.22]
At least once a week			0.74	[0.51–1.07]
Frequency of reading Newspaper				
Not at all			1	[1]
Less than once a week			1.15	[0.75–1.77]
At least once a week			2.89	[0.79–4.45]
Region				
North central			0.49**	[0.31–0.78]
North east			0.54**	[0.37–0.79]
North west			1	[1]
South east			0.42**	[0.21–0.81]
South south			0.26***	[0.14–0.49]
South west			0.29***	[0.16–0.54]

*OR* Odds Ratio, *aOR* Adjusted Odds Ratio, *CI* Confidence interval; [in square brackets; 1 = reference; *p < 0.05, **p < 0.01, ***p < 0.001]

### Ethical approval

The 2018 NDHS was reviewed and approved by the National Health Research Ethics Committee of Nigeria (NHREC) and ICF’s Institutional Review Board. Demographic and Health Surveys follow ethical standards outlined by the U.S. Department of Health and Human Services regulations for the respect of human subjects. Detailed information on the ethical procedures observed by the DHS program can be accessed via https://goo.gl/ny8T6X. Since this study was based on data that is publicly available, no further ethical requirements were required.

## Results

### Residential status, socio-demographic characteristics and ITN utilization among pregnant women

Overall, 4066 pregnant women took part in the survey, however, 2909 pregnant women participated in the study because their household owned at least one ITN. Those living in rural Nigeria were more than twice (69.1%) the proportion of women in urban parts of the country (30.9%). Nearly nine out of ten of the rural residents slept under ITN (86.1%). Most of the pregnant women were 25–29 years old (27.0%) only 1.6% aged 45–49. Most of those aged 15–19 indicated that they used ITN (87.2%). More than half of the pregnant women had no formal education (55.0%) and 88.1% of them used ITN. ITN utilization was high among poorer (88.9%) and poorest (88.5%) women. Most of the pregnant women were members of the Islam religion (73.5%) and ITN utilization was well pronounced among women of the Traditionalist/Other religion (91.5%). Almost all the pregnant women were married (97.5%) and the majority (82.6%) of these married women indicated that they utilized ITN. Husbands/partners alone took healthcare decisions of nearly seven out of ten women (68.7%) and most of this same category used ITN (85.3%).

Most of the women indicated that they wanted their pregnancies at the time they got pregnant (89.0%) and more than half of them utilized ITNs (83.8%). Almost all the women were not subscribed to the health insurance (97.2%), and 82.5% of this same category reported that they utilized ITNs. More than half of them reported that they were not listening to radio at all (53.7%) and 85.2% of this category of women utilized ITNs. Nearly six out of ten of the women were not watching television at all (65.8%) and 87.4% of them utilized ITNs. Women who were not reading newspapers stood at 91.6%, however 84.8% who were reading newspaper at least once a week utilized ITNs as shown in Table 1. Nearly half of the women were residing in north-west region (49.5%) and 89.4% of this same women utilized ITN.

### Binary logistic regression on residential status and ITN utilization

Table 2 presents the outcome of the binary logistic regression on residential status and ITN utilization among pregnant women of Nigeria. Those living in rural locations had two times higher odds of utilizing ITN compared to pregnant women in urban locations [cOR = 2.17, CI = 1.66–2.84]. After controlling for socio-demographic characteristics, the adjusted model similarly indicated higher odds of ITN utilization among pregnant women who reside in rural locations compared to those in urban residents [aOR = 1.18, CI = 1.05–1.24]. Pregnant women aged 40–44 years had lower odds of ITN utilization compared to those aged 15–19 (reference category) [aOR = 0.63, CI = 0.44–0.92].

Poorer pregnant women had higher odds of ITN utilization compared with poorest pregnant women [aOR = 1.09, CI = 1.04–1.32]. Women who wanted their pregnancies later than the time the pregnancy occurred had lower odds of using ITNs than those who wanted their pregnancies the time they occurred [aOR = 0.66, CI = 0.46–0.97]. Pregnant women who were listening to radio at least once a week were more likely to use ITN compared to those who were not listening at all [aOR = 1.32, CI = 1.07–1.74]. Relative to women who reside in North west region, women of all other regions were less likely to use ITN especially compared with those in the south [aOR = 0.26, CI = 0.14–0.49] and south west [aOR = 0.29, CI = 0.16–0.54].

## Discussion

This study investigated variation in ITN utilization between rural and urban pregnant women in Nigeria. The study revealed that pregnant women who live in rural locations had higher odds of utilizing ITN compared with those in urban locations. This supports previous findings that ITN utilization is more prevalent in rural Nigeria compared to the urban settings [38]. However, most previous studies have reported contrary finding from Nigeria [39] and other SSA countries, such as Ghana [36], Equitorial Guinea [40], Senegal [41] and Ethiopia [33]. Even where more rural have possessed ITNs in Nigeria, utilization has been reported to be low [29, 42].

In spite of the consistency in high prevalence of ITN utilization among urban women as aforementioned, some studies from Ghana are consistent with our outcome [43, 44]. The finding is also consistent with evidence from the 2010 and 2015 Nigeria Survey reports [14, 15] and national malaria operational plan [45]. Although countless advantageous factors enable urban residents to possess and utilize ITN [39, 41], this finding is plausible as most previous ITN initiatives have targeted rural residents as a way of bridging this gap [27, 46]. Rural based campaigns have also taken place such as the one that occurred in the Enugu State [47]. Not notwithstanding, some urban locations with malaria burden such as the Federal Capital of Abuja have also been targeted with such mass campaigns by the NMEP [19]. The evidence from this study may imply that such initiatives are gradually yielding the desirable outcome.

Although evidence suggest that urban locations are less conducive for vector species [48, 49], some urban locations tend to have high malaria cases than nearby rural settings and results from widespread mosquito breeding sites such as urban agriculture, tyre tracks, and ditches [50]. Moreover, peri-urban residents with low socio-economic status also tend to have high prevalence of malaria [51]. At the convergence of the current finding and these previous reports is the realization that, ITN campaigns ought to consider peri-urban locations and urban centres where possible breeding sites are immanent.

Pregnant women aged 40–44 years had lower odds of ITN utilization compared with those aged 15–19. This observation, however, varies from some earlier findings from Nigeria and elsewhere [4, 29]. Ankomah et al*.* [29] asserted that younger women may have lower chances to ITN utilization because they have lower chance of previous pregnancy, which would have granted them opportunity to be educated about the need to utilize ITN. Meanwhile, the finding concurs with one study that assessed awareness, ownership and use of treated ITNs in the Ekiti State of Nigeria [25]. It is worth noting that young women are likely to have limited pregnancy experience compared with older women, and as such may be scared and thereby remain committed to all measures that can protect them and their fetus including consistent ITN utilization.

Poorer women had higher odds of ITN utilization. Most poor women in Nigeria are rural residents [39, 52], and this suggests that the characteristics of these women are in tandem with the features of rural women as far as ITN utilization is concerned. Baume and Franca-Koh [44] also recount that in Ghana, women with lower socio-economic status (SES) had higher chances of utilizing ITNs. The poor may be using ITNs as their only measure against mosquito bite. Nonetheless, rich women may be in households and environments that are well protected against mosquitoes, or use other means to prevent mosquito bites, thereby rendering ITN utilization needless [32].

Women who wanted their pregnancies later than the time the pregnancies occurred had lower odds of using ITNs compared with those who wanted their pregnancies when they occurred. Pregnancy intendness or otherwise appear to have several implications on precautionary measures taken by pregnant women. Compared with women whose pregnancies occur unexpectedly, utilization of ITN is more apparent among women with intended pregnancy. This is because unintended pregnancy (i.e. pregnancies occurring earlier or later than expected or not needed at all) contributes to an increase in psychosocial distress and stress levels thereby compromising the ability of a woman to exercise the necessary precautionary measures [53, 54]. It is however, noteworthy, that several other factors may contribute to less ITN use other than psychosocial distress and stress levels. In Rwanda, Thogarapalli et al*.* [55] similarly observed that women with unintended pregnancies had reduced chances of utilizing ITNs. Women with unintended pregnancies in Nigeria may require further encouragement and education about ITN use.

Pregnant women who listened to radio at least once a week had higher odds of ITN utilization than those who did not listen at all. Radio has earlier been identified as an effective mass media option for delivering ITN messages in Nigeria [29]. Radio is cost effective and has the potential for reaching large audience in a language that is easily understood by the target audience [56]. The observations made between radio and ITN utilization could imply that radio is the principal medium for ITN advocacies.

Relative to North West region, women in all other regions had lower odds of ITN utilization especially those in the South West. It is worth noting that mass ITN campaigns have taken place throughout the country [13]. The variation across the regions may be attributable to two main explanations; behavioural issues and variation in mass campaign strategies across states. As espoused by the Health Belief Model, adoption of a health protective behaviour, ITN use in this case, is a function of perceived risk of contracting malaria from mosquito bites and perception of severity/fatality of malaria [57]. In the case of malaria, these thoughts vary by region in the case of Nigeria as revealed by a study that investigated knowledge and preventive precautions of malaria [58]. Second, due to variation in temperature, geographical terrain, environmental factors and malaria prevention strategies across regions [58–60], there may be variations in intensity of mosquitoes across regions. In that instance, ITN use is expected to be high in highly mosquito prevalent regions relative to less prevalent ones. With these, a thorough reflection of mass campaigns and anti-malarial initiatives to increase ITN availability in each household should be a topmost priority. This is because almost all subgroups identified in the study recorded ITN usage above 60%, which is closer to the target of 80%.

### Strengths and limitations

This study presents evidence from the 2018 Demographic and Health Survey data, which is the most current nationally representative data. The representative nature of the survey, the dual stage sampling approach and rigour of the analyses reinforce the validity of the findings. Amidst these strengths, the study has some limitations. There is the temptation for some women to be coerced by social desirability bias depending on the thoughts or impression of their immediate environment (e.g. partner, parents and friends) about ITN. It is worth interpreting our results in light of the cross-sectional nature of the survey. Due to the seasonality of malaria, the timing of the survey might have affected the findings.

## Conclusion

The study showed high ITN utilization among rural pregnant women in Nigeria. Socio-demographic predictors of ITN utilization include age, education, wealth, religion, pregnancy intendness and exposure to radio. The findings have revealed dimensions worth acknowledging in order to enhance the prospects of achieving national anti-malarial initiatives, such as the NSMSP and the NMEP. Since possession of ITN is not a guarantee for utilization, women in urban locations need constant reminder of ITN use through messages delivered at ANC and radio advertisements. Successful ITN interventions will be the ones that can address the contextual bottlenecks of ITN utilization for rural and urban women in Nigeria concurrently. The study has revealed that in addition to planning interventions along the lines of residence (rural–urban), other essential characteristics such as age, education wealth, religion, pregnancy intendness and women’s exposure to radio should guide future interventions that intend to upsurge ITN utilization among pregnant women in Nigeria. Subsequent mass ITN campaigns and responsive interventions ought to take cognizance of subgroups with suboptimal ITN use such as those in south-west and south regions. Pragmatic steps should also be taken to increase the availability of ITN in households since there is a moderately high use in households with at least one ITN in Nigeria.

## Data Availability

Data used for the study is freely available to the public via https://dhsprogram.com/data/available-datasets.cfm.

## Abbreviations

aOR: Adjusted Odds Ratio
ANC: Antenatal Care
CI: Confidence Interval
cOR: Crude Odds Ratio
DHS: Demographic and Health Survey
ITN: Insecticide Treated Net
NSMSP: National Strategic Malaria Strategic Plans
NHREC: National Health Research Ethics Committee of Nigeria
NMEP: National Malaria Elimination Programme
NDHS: Nigeria Demographic and Health Survey
NPC: National Population Commission
RBM: Roll Back Malaria
SSA: Sub-Saharan Africa
VIF: Variance Inflation Factor
